# Can Optimism, Pessimism, Hope, Treatment Credibility and Treatment Expectancy Be Distinguished in Patients Undergoing Total Hip and Total Knee Arthroplasty?

**DOI:** 10.1371/journal.pone.0133730

**Published:** 2015-07-27

**Authors:** Tsjitske M. Haanstra, Claire Tilbury, Steven J. Kamper, Rutger L. Tordoir, Thea P. M. Vliet Vlieland, Rob G. H. H. Nelissen, Pim Cuijpers, Henrica C. W. de Vet, Joost Dekker, Dirk L. Knol, Raymond W. Ostelo

**Affiliations:** 1 Department of Epidemiology and Biostatistics and the EMGO Institute for Health and Care Research, VU University Medical Centre, Amsterdam, The Netherlands; 2 Department of Orthopaedics, Leiden University Medical Center, Leiden, The Netherlands; 3 Musculoskeletal Division, The George Institute for Global Health, The University of Sydney, Sydney, New South Wales, Australia; 4 Department of Orthopaedics, Rijnland Ziekenhuis, Leiderdorp, The Netherlands; 5 Department of Clinical Psychology and the EMGO Institute for Health and Care Research, VU University Amsterdam, The Netherlands; 6 Department of Rehabilitation Medicine and the EMGO Institute, VU University Medical Centre, Amsterdam, The Netherlands; 7 Department of Epidemiology and Biostatistics, VU University Medical Centre, Amsterdam, The Netherlands; 8 Department of Health Sciences and the EMGO Institute for Health and Care Research, VU University, Amsterdam, The Netherlands; Tilburg University, NETHERLANDS

## Abstract

**Objectives:**

The constructs optimism, pessimism, hope, treatment credibility and treatment expectancy are associated with outcomes of medical treatment. While these constructs are grounded in different theoretical models, they nonetheless show some conceptual overlap. The purpose of this study was to examine whether currently available measurement instruments for these constructs capture the conceptual differences between these constructs within a treatment setting.

**Methods:**

Patients undergoing Total Hip and Total Knee Arthroplasty (THA and TKA) (Total N = 361; 182 THA; 179 TKA), completed the Life Orientation Test-Revised for optimism and pessimism, the Hope Scale, the Credibility Expectancy Questionnaire for treatment credibility and treatment expectancy. Confirmatory factor analysis was used to examine whether the instruments measure distinct constructs. Four theory-driven models with one, two, four and five latent factors were evaluated using multiple fit indices and Δχ^2^ tests, followed by some posthoc models.

**Results:**

The results of the theory driven confirmatory factor analysis showed that a five factor model in which all constructs loaded on separate factors yielded the most optimal and satisfactory fit. Posthoc, a bifactor model in which (besides the 5 separate factors) a general factor is hypothesized accounting for the commonality of the items showed a significantly better fit than the five factor model. All specific factors, except for the hope factor, showed to explain a substantial amount of variance beyond the general factor.

**Conclusion:**

Based on our primary analyses we conclude that optimism, pessimism, hope, treatment credibility and treatment expectancy are distinguishable in THA and TKA patients. Postdoc, we determined that all constructs, except hope, showed substantial specific variance, while also sharing some general variance.

## Introduction

Growing evidence supports the importance of psychological constructs in predicting outcomes of medical treatment including surgery [1–7]. Usually epidemiological studies investigating the relationship between psychological factors and outcome of treatment restrict their assessment to one or two psychological questionnaires. However, in order to disentangle their unique contribution to outcome, the roles of separate constructs need to be explored simultaneously [8]. It is therefore necessary that the instruments that aim to measure these constructs are able to discriminate between them.

Much attention has been given to the future oriented constructs ‘optimism’, and ‘hope’ [1,9]. Both these constructs reflect expectancies about one’s future. More specifically, optimism has been defined as “generalized positive outcome expectancies” [10] and hope as “a cognitive set that is based on a reciprocally derived sense of successful agency (goal directed determination) and pathways (planning of ways to meet goals)” [11]. Theory suggests that a hopeful person is more explicitly concerned with self-initiated actions that will enable him to achieve a favourable future while an optimistic person believes that somehow (through either internal or external factors) his future will be successful [12]. Substantial empirical work investigating optimism and hope has been done within mental health settings. But evidence suggests these constructs may also be related to outcomes in medical treatments like surgery. For example: optimism explains close to 10% of the variance in post-surgical pain after total hip arthroplasty (THA) and total knee arthroplasty (TKA) [13].

Besides these general future oriented constructs interest in treatment specific psychological constructs like ‘treatment expectancy’ and ‘treatment credibility’ has also grown [14]. Treatment expectancy is defined as “improvements that clients believe will be achieved” and treatment credibility as “how believable, convincing and logical the treatment is” [15]. Conceptually, expectations for a given treatment may develop (at least partly) from how credible the treatment seems. Both these constructs may be related to treatment outcomes. For example it was found that expectancies about treatment outcome help predict return to work outcomes [16].

While the abovementioned psychological constructs are grounded in different theoretical models, some studies have hypothesized that there is some conceptual overlap between them [1,10–12,17–21]. Optimism, hope, treatment credibility and treatment expectancy have for instance all been conceptualized as an anticipatory state and beliefs about the future [11,12,22,23]. Others have emphasized the conceptual differences between the constructs. Some suggest hope is an emotional state, while optimism is a cognitive state [24]. Treatment credibility also has been defined as a cognitive concept, whereas treatment expectancy as a more affective or emotional concept, similar to hope [15]. Furthermore, treatment credibility and treatment expectancy are conceptualized to be situational (i.e. treatment specific), in contrast to hope and optimism which are dispositional [15,25].

Multiple studies have empirically explored the distinction between the constructs optimism (and pessimism) and hope. A recent meta-analysis concluded that these constructs are positively associated but not redundant (rho <0.8) and that hope and optimism have differential relationships with outcomes like well-being or personality [9]. Treatment expectancy and treatment credibility however have not been included in studies examining the distinctiveness of future oriented constructs, yet. It may be that in medical situations like upcoming elective surgery patients answer items belonging to the hope and optimism questionnaires more in a situational way, referring to their treatment or illness. Consequently in medical treatments, and more specifically in the invasive treatments like surgery, optimism and hope could possibly show similarities to treatment credibility and treatment expectancy.

This study aims to examine whether the instruments for optimism, pessimism, hope treatment credibility and treatment expectancy measure distinct constructs in a population of patients scheduled for THA or TKA.

## Materials and Methods

### Participants and procedures

This study was part of a larger prospective cohort study on the outcomes of THA and TKA. It included consecutive patients undergoing a primary THA or TKA because of osteoarthritis in the Rijnland Hospital in Leiderdorp, the Netherlands between October 2010 and September 2012. Assessments were done pre-operatively and 12 months after surgery. Between July 2011 and September 2012 a subgroup of participants received additional questionnaires concerning optimism, hope and expectancies pre-operatively. For the present analysis pre-operative data of this subgroup were used. The larger study, as well as the extension for the subgroup was approved by the Medical Ethical Committee of the Rijnland General Hospital, Leiderdorp, the Netherlands (registration number 10/07). All participants gave written informed consent.

### Measurement

One day prior to surgery all participants completed a questionnaire including sociodemographic, disease characteristics, Quality of Life and the Life Orientation Test-Revised (LOT-R), the Hope Scale (HS) and the Credibility Expectancy Questionnaire (CEQ). Demographic characteristics included: age (years), sex and education level. Disease characteristics pain and functioning were measured using the Pain and ADL subscales of the HOOS (for THA patients) [26] and KOOS (for TKA patients) [27] questionnaires. Quality of Life was measured using the SF-36 questionnaire [28] from which mental component scores (MCS) and physical component scores (PCS) were derived.

### Optimism (and pessimism)

The Life Orientation Test- Revised [20] is a 10 item self-reported questionnaire that aims to measure optimism. The questionnaire consists of 3 positively formulated items (e.g. I’m always optimistic about my future), 3 negatively formulated items (e.g. I rarely count on good things happening to me) and 4 filler items (e.g. It’s easy for me to relax), all items are answered on a 5 point Likert-type scale. The LOT-R was originally developed by Scheier and Carver in 1994 [20] who called the LOT-R a unidimensional questionnaire in which the observed variables represent one latent factor called trait optimism. However others have argued that the items in the LOT-R represent two latent factors namely optimism and pessimism [29,30]. Hence, sumscores range from 3–15 when two subscale scores are calculated or from 3–30 when one total score is calculated. The factor structure of the Dutch version of the LOT-R was tested recently [31]. Results showed that the two factor model had the best fit.

### Hope

The Hope Scale consists of 12 items of which 4 items measure ‘pathways’ (e.g. There are lots of ways around the problem), 4 items measure ‘agency’ (e.g. I meet the goals that I have set for myself) and 4 are filler items (e.g. I worry about my health) [11]. All items are answered on an 8 point scale with two anchors (1 = totally disagree and 8 = totally agree).The hope scale is considered to be a unidimensional scale in which agency and pathways together represent the construct ‘trait hope’. Analysis of the Dutch version of the HS has shown good model fits for a one factor structure [32]. Hence, a sumscore which ranges from 8–64 points is derived by summing the 8 items of the HS.

### Treatment credibility and treatment expectancy

The Credibility Expectancy Questionnaire is a self-reported six item questionnaire that aims to measure treatment credibility and expectancy for improvement. Originally it was developed by Devilly et al in 2000 [15], and validated in several groups. The Dutch translation was done by Smeets et al in 2008 [14]. In both the original and the Dutch version three items (e.g. at this point, how successfully do you think the surgery will be in reducing your complaints) were found to load on the credibility factor and three items (e.g. at this point, how much do you really feel that the surgery will help to reduce your complaints) on the expectancy factor. Introductory instructions tell the patient that beliefs about how well the therapy might help contain both thoughts and feelings about the therapy and that these may be the same or different [14]. Items 1 to 3 and 5 are answered on a scale ranging from 1 (not at all) to 9 (very much), Items 4 and 6 are answered on a 0 (not at all) to 100% (very much). In accordance with Smeets et al scores on item 4 and 6 were transformed with a minimum of 1 and a maximum of 9, and a sum score was formed for each factor ranging from 3 to 27.

### Statistical analysis

Confirmatory factor analysis (CFA) for ordered categorical items was used to examine whether the constructs optimism (LOT-R), pessimism (LOT-R), hope (HS), treatment credibility (CEQ credibility subscale) and treatment expectancy (CEQ expectancy subscale), are distinguishable. Because observed variables were all answered on ordinal scales, a matrix based on polychoric correlations was used for CFA. Negatively formulated items of the LOT-R were reverse scored prior to entry into the CFA models. Analyses were conducted using the weighted least squares mean and variance adjusted estimator (WLSMV) in Mplus 6.12. For the total group of THA and TKA patients four theory-driven models with five, four, two and one latent factors, in which the factors were allowed to correlate within the CFA models, were evaluated using multiple fit indices and compared using Δχ^2^ tests [33]. The following fit indices and thresholds were used to denote a satisfactory model: Tucker-Lewis index (TLI) >0.95; comparative fit index (CFI) >0.95 and the root mean square error of approximation (RMSEA) <0.06 [34]. A significant Δχ^2^ test indicates that the model with the smallest χ^2^ (in this case the least stringent model) has a significantly better fit.

Model 1 hypothesised a full differentiation between the five constructs treatment credibility, treatment expectancy, hope, optimism and pessimism. Thus items of each construct was forced to load on a separate factor. Model 2 hypothesised a differentiation between four constructs; the treatment credibility, treatment expectancy and hope items were still forced to load on separate factors, but in this model the optimism and reverse-scored pessimism items were forced to load on one factor as it is controversial whether LOT-R has a uni- or bidimensional structure [29,30]. Model 3 hypothesised a two factor structure in which the optimism, pessimism and hope (LOT-R and HS) items were forced to load on one factor representing ‘generalized positive beliefs about the future’ and the treatment credibility and treatment expectancy (CEQ) items were forced to load on one factor representing ‘treatment specific beliefs about the future’. This model was tested because of the theoretical plausibility that patients may have general and situational, in this case treatment specific, beliefs about the future. Model 4 hypothesised that treatment credibility, treatment expectancy, hope, optimism and pessimism items load on a single underlying latent factor. This model was tested because when it is assumed that optimism, pessimism, hope, treatment credibility and treatment expectancy are not distinguishable at all, the data should fit this one factor model. If necessary (eg because of ambiguities or high correlations between factors) post-hoc models were tested. Guttman’s lambda 2 was used to determine internal consistency reliability of each subscale. A value > 0.7 was considered indicative of good internal consistency reliability [35,36]. All the analyses above were done using the total sample of THA and TKA patients.

When using the same questionnaire in different groups Factorial Invariance (FI) should be established to show that the items of the questionnaire measure the particular latent construct similarly across groups. In our study both TKA and THA patients were included, and as patients with scheduled for knee arthroplasty may face different difficulties to patients scheduled for hip arthroplasty, the constructs measured in this study may also have different meanings for these groups.

Assessing factorial invariance involves a process of comparing the fit indices for a series of models with increasingly stringent constraints on the relationships between the model parameters. The best-fitting model for the total sample (TKA and THA) identified in the previous analysis was assessed in multigroup CFA’s to test for factorial invariance across the TKA and THA groups[37]: Four multigroup CFA models with increasingly stringent model constraints were tested (Table 1):

- A baseline model (configural invariance): in which only the factor structure (number of factors and the pattern of the free and fixed loadings) was constrained to be equal across groups. In this model no equality constraints were imposed on the intercepts and factor loadings.
- A weak FI model: in which the factor structure and factor loadings were constrained to be equal across groups, intercepts were allowed to vary among groups and factor variances were fixed to one in both groups.
- A strong FI model: in which factor structure and loadings and intercepts (thresholds) were constrained to be equal across groups.
- A strict FI model: in which factor structure, factor loadings, intercepts and residual variances were constrained to be equal across groups.

**Table 1:** Levels of factorial invariance

*FI Models*	Model parameters constrained to be equal across groups
**No FI**	None
**Weak FI**	Factor loadings
**Strong FI**	Factor loadings and item intercepts (thresholds)
**Strict FI**	Factor loadings and item intercepts (thresholds) and residual item variances/covariances

To evaluate the degree of measurement invariance, the recommendations by Cheung and Rensvold [38] were followed, which state that the null hypothesis (invariance) is kept if the incremental change in comparative fit index (CFI) is equal to or smaller than 0.01 [38]. Acceptance of the strong or the strict invariance model was sufficient to assume that the measurement instruments used measure the same constructs in all participants (both THA and TKA).

Missing data were incorporated by using the default option available in Mplus. For WLMSV estimation, Mplus computes polychoric correlations based on pairwise present data between two variables, treating missing data as missing completely at random (MCAR). Under MCAR, the missingness is assumed to occur entirely at random and not depend on observed covariates or on the response itself.

## Results

### Characteristics of the sample and internal consistency reliability of the subscales

A total of 745 patients were admitted for THA and 614 patients were admitted for TKA from October 2010 to September 2012. Of these, 420 THA (63.2%) and 395 TKA (65.9%) patients consented to participate and completed the surveys. A subgroup of 184 THA and 191 TKA patients, the ones enrolled in the study between July 2011 and September 2012, received additional questionnaires including the LOT-R, the HS and the CEQ. Of these, 14 had missing responses on all items and were therefore excluded, leaving in total 361 patients for analysis (182 THA, 179 TKA). Characteristics of the subgroup of participants that completed the additional questionnaires and mean scores (sd) on the subscales of these questionnaires are presented in Table 2 for THA and TKA groups separately. In both TKA and THA groups the majority of patients were females, the mean age was 67 years for both groups. The mean pain score was 41.9 for THA patients and 39.7 for TKA patients. The mean functioning score (HOOS/KOOS ADL) was 43.8 for THA patients and 45.6 for TKA patients. THA patients on average scored 23.7 on the credibility and 22.5 on the expectancy subscale of the CEQ, TKA patients scored 23.5 and 22.1 on these subscales respectively. HS scores were 43.2 for THA and 41.3 for TKA patients. Optimism was scored 9.9 for THA patients and 10.0 for TKA patients, Pessimism scores were 10.8 for THA and 10.4 for TKA patients. Internal consistency reliability (lambda 2) of each of the subscales was acceptable (Table 2). For 14 patients responses on all items were missing and therefore they were excluded from analysis. All the questionnaire items had missing responses, though in most items < 7% responses were missing. An exception was one of the HS items which had 42% missing responses (item 6) due to a printing error in the questionnaire. The amount of data in the pairwise coefficients ranged between 0.57 and 0.98.

**Table 2:** Characteristics of the sample included in this study and lambda 2 values for the subscales included in the Confirmatory Factor Analysis.

	*Total hip arthroplasty (N = 182) mean (SD)/ %*	*Total knee arthroplasty (N = 179) mean (SD)/%*	*Total sampleN = 361 Mean (SD)/%*	Gutmann’s lambda 2 for the total sample
**Gender % female**	58.1%	71.6%	64.8%	-
**Age**	67.1 (9.9))	67.6 (9.3)	67.4 (9.6)	-
**Education level**				-
*Low*	33.7%	50.3%	42.0%	
*Medium*	32.0%	31.2%	31.6%	
*High*	34.3%	18.5%	26.4%	
**BMI**	27.5 (4.7)	29.5 (4.8)	28.5 (4.9)	-
**SF-36 Physical summary scale (range 0–100)**	38.8 (7.1)	39.1 (7.7)	38.9 (7.4)	-
**Sf-36 Mental summary scale (range 0–100)**	51.8 (10.7)	52.2 (11.2)	52.0 (10.9)	-
HOOS/KOOS* pain (range 0–100)	41.9 (17.8)	39.7 (16.7)	40.6 (17.2)	**-**
HOOS/KOOS* Activities of Daily Living (range 0–100)	43.8 (17.6)	45.6 (17.6)	44.7 (17.5)	-
**CEQ Credibility (range 3–27)**	23.7 (3.0)	23.5 (3.1)	23.6 (3.1)	0.714
**CEQ Expectancy (range 3–27)**	22.5 (3.0)	22.1 (3.0)	22.3 (3.0)	0.779
**HS Hope (range 8–64)**	43.2 (11.6)	41.3 (11.8)	42.2 (11.7)	0.941
**LOT-R Optimism (range 3–15)**	9.9 (2.9)	10.0 (2.8)	9.9 (2.8)	0.834
**LOT-R Pessimism, reverse scored (range 3–15)**	10.8 (2.8)	10.4 (2.7)	10.6 (2.8)	0.709

*The THA patients completed the HOOS questionnaire and the TKA patients completed the KOOS questionnaire.

HOOS = the Hip injury and Osteoarthris Outcome Score, KOOS = the Knee injury and Osteoarthritis Outcome Score, CEQ = Credibility Expectancy Questionnaire, HS = Hope Scale, LOT-R = Life Orientation Test Revised.

### Confirmatory factor analysis

Table 3 shows the model fit indices for the five, four, two and one factor models, as well as Δχ^2^ tests comparing the five factor model with the four factor model, the four factor model with the two factor model, and the two factor model with the one factor model. The five factor model showed fit indices that satisfied the cut-off criteria determined by Hu and Bentler [34], whilst the models with four, two and one latent factor did not satisfy these criteria. Further, Δχ^2^ tests also indicated that the four factor model fit significantly worse than the five factor model, the two factor model fit significantly worse than the four factor model and the one factor model fit significantly worse than the two factor model. Thus, of the four models tested the five factor model is to be preferred based on all fit indices.

**Table 3:** χ^2^ difference tests and model fit indices for the models tested for the total group (THA and TKA).

	*χ* ^*2*^ *(df)*	*P-value*	*Δχ* ^*2*^ *(df)*	*P-value*	*TLI*	*CFI*	*RMSEA*
**Five factor model**	400.9 (160)	<0.01	237.1 (4) ^$^	<0.01	0.981	0.984	0.065
**Four factor model**	1121.8(164)	<0.01	86.7 (5) ^#^	<0.01	0.927	0.937	0.127
**Two factor model**	1220.4(169)	<0.01	271.1 (1) §	<0.01	0.922	0.930	0.131
**One factor model**	3081.9 (170)	<0.01			0.785	0.807	0.218

$ five factor model compared to four factor model model

# four factor model compared to two factor model

§ two factor model compared to one factor, TLI = Tucker-Lewis Index, CFI = comparative fit index, RMSEA = root mean square error of approximation.

**Fig 1:**
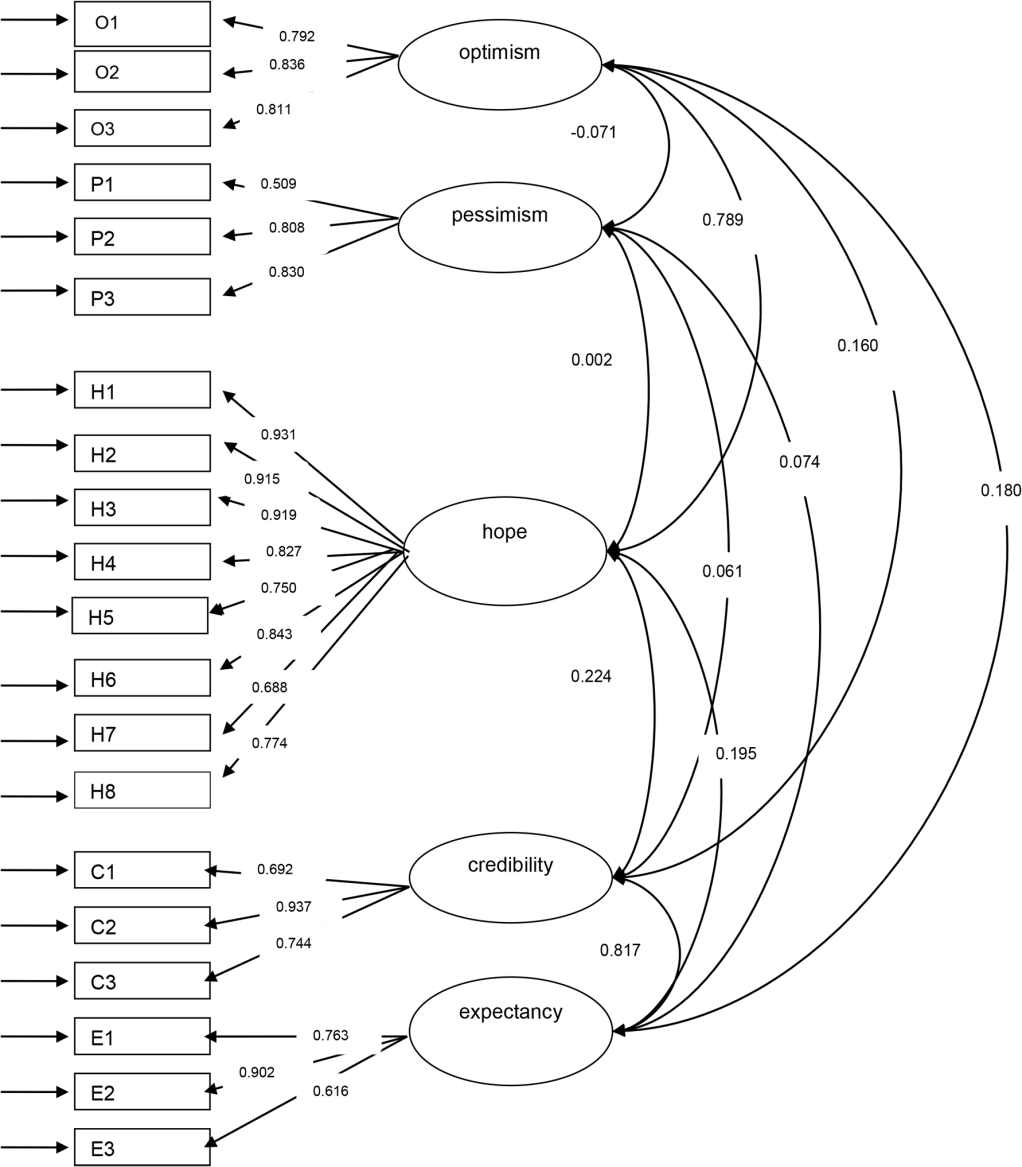
Path diagram and standardized factor loadings and correlations between factors for the 5 factor model. O1—O3 = LOT-R optimism items 1 to 3,P1—P3 = LOT-R reverse scored pessimism items 1 to 3,H1—H8 = ADHS hope items 1 to 8,C1—C3 = CEQ credibility items 1 to 3,E1—E3 = CEQ expectancy items 1 to 3 ovals represent latent factors, squares represent observed variables, factor loadings are represented by the arrows between ovals and squares and correlations between factors are represented by the arrows between the ovals.

The five factor model including the standardized factor loadings and correlations between factors is presented in Fig 1. In this five factor model a very strong correlation was seen between the treatment credibility and treatment expectancy factors, and a strong correlation between the optimism and hope factors.

Factorial Invariance testing (see Table 4) showed that the baseline model was well-fitting and thereby supported configural invariance. For the increasingly stringent models none of the subsequent null-hypotheses of measurement invariance were rejected using the recommendations of Cheung and Rensvold [38] which state that the null hypothesis (invariance) is not rejected if the incremental change in CFI is equal to or smaller than 0.01. Thus, strict invariance could be supported.

**Table 4:** Model fit indices of the multigroup models for factorial invariance testing across THA and TKA.

*Factorial Invariance models*	*Χ* ^*2*^ *(df)*	*P value*	*TLI*	*CFI*	*RMSEA*
**Baseline model (configural invariance)**	640.1 (415)	<0.01	0.986	0.985	0.055
**Weak Invariance**	653.6 (430)	<0.01	0.987	0.985	0.054
**Strong Invariance**	672.4 (405)	<0.01	0.983	0.982	0.060
**Strict Invariance**	718.6 (425)	<0.01	0.983	0.981	0.062

Because of the very strong correlation between treatment expectancy and treatment credibility, and between hope and optimism, three post-hoc analyses were performed. A four factor model with separate factors for hope, optimism and pessimism but in which expectancy and credibility items were forced to load on one factor (S1 Fig) showed fit indices equal to the five factor model (TLI = 0.98, CFI = 0.98, RMSEA = 0.06, χ^2^ (df) = 412.5 (164)) The Δχ^2^ test indicated that the four factor model fit significantly worse than the five factor model (Δχ^2^ (df) = 16.1 (4) p<0.01)).A four factor model with separate factors for treatment expectancy, treatment credibility and pessimism but in which optimism and hope were forced to load on one factor (S2 Fig), had a slightly worse fit compared to the five factor model (TLI = 0.97, CFI = 0.97, RMSEA = 0.08, χ^2^ (df) = 568.7 (164)). The Δχ^2^ test indicated that the four factor model fit significantly worse than the five factor model (Δχ^2^ (df) = 102.7 (4) p<0.01).

Further, a bifactor model in which (besides the 5 separate factors) there is a general factor (gf) that is hypothesized to account for the commonality of the items of the 5 separate constructs was tested (S3 Fig). This bifactor model showed better fit indices than the 5 factor model and the four factor model in which the expectancy and credibility items were forced to load on one factor (TLI = 0.99, CFI = 0.99, RMSEA = 0.06, χ^2^ (df) = 304.6 (140)). The Δχ^2^ test indicated that the bifactor model fit statistically significantly better than the five factor model (Δχ^2^ (df) = 86.8 (20) p<0.01). For the bifactor model we calculated the proportion of variance accounted for by all factors (ω_k_), the proportion of variance accounted for by the general factor (ω_H_). For each of the 5 separate factors we calculated the proportion of variance unique from the general factor (ω_Nk_) (for example see [39,40]). For these unique proportions of variance a value of ω_Nk_ ≥ 0.30 was regarded as substantial, a value of 0.20 ≤ ω_Nk_ <0.30 was regarded as moderate, and a value of ω_Nk_ <0.20 was regarded low [40]. Table 5 shows that the total amount of variance accounted for by all factors is large (0.94). Also, a substantial amount of variance of all factors (ω_k_) is accounted for by variation in the general factor (0.79). This suggest that all items indeed measure a common construct. However the specific factors differ in how much variance they account for unique from the general factor. Treatment expectancy, treatment credibility, optimism and pessimism explain a substantial amount of variance unique from the general factor, however hope does not explain a substantial amount of variance unique from the general factor (Table 5).

**Table 5:** The proportion of variance explained by all factors (ω_k_), the proportion of variance of the total scale explained by the general factor (ω_H_) and the proportion of variance of the separate constructs explained by the specific factors (ω_Nk_).

*Scale*	*ω*_*k*_	*ω*_*H*_	*ω*_*Nk*_
**Total model (general factor)**	0,942	0,787	
**Separate constructs**			
Treatment credibility	0,838		0,789
Treatment expectancy	0,809		0,782
Hope	0,956		0,015
Optimism	0,857		0,329
Pessimism	0,769		0,769

## Discussion

This study examined whether the existing instruments for optimism, pessimism, hope, treatment credibility and treatment expectancy measure distinct psychological constructs in patients undergoing TKA or THA. Because it was not our purpose to develop new instruments or to revise the existing ones, we chose a confirmatory approach (CFA) in all our analyses instead of an exploratory approach (EFA). Moreover, we aimed to use all instruments in the same way as they are currently utilized in research and practice and therefore did not delete items with low factor loadings.

The results of the theory driven CFA showed that a five factor model in which optimism (LOT-R subscale optimism), pessimism (LOT-R subscale pessimism), hope (HS), treatment credibility (CEQ subscale credibility) and treatment expectancy (CEQ subscale expectancy) had the most optimal fit. However, there were two interesting observations. First, a strong correlation (r = 0.82) was observed between expectancy and credibility. Therefore a post-hoc analysis was performed in which a four factor model in which expectancy and credibility were forced to load on one factor was tested. Although fit indices were very similar as the five factor model, the Δχ^2^ test indicated the five factor model was the preferred model. Earlier studies found moderate to very high correlations between expectancy and credibility (r = 0.56 [14], r = 0.68 [15] and r = 0.83 [15]), though exploratory as well as confirmatory factor analyses suggest expectancy and credibility are two separate factors [14,15]. Although the current study cannot provide the definite answer regarding the distinctiveness of the constructs treatment expectancy and treatment credibility in patients undergoing THA or TKA, it seems reasonable that the a-priori, theory driven 5 factor model is preferred. Additionally the five factor model is also supported by the results of those earlier studies on the constructs expectancy and credibility. Nevertheless, future studies should investigate the factorial structure of the CEQ and the distinctiveness of the constructs treatment credibility and treatment expectancy to determine if our findings are replicable or unique to our study sample.

Secondly, a strong correlation was also observed between optimism and hope (r = 0.79). A priori we hypothesized that these two factors would be correlated but still distinct because both are defined as general future oriented constructs but do have considerable theoretical differences, though we did not expect such a strong correlation. We therefore also performed a post-hoc analysis to investigate the influence of the strong correlation between hope and optimism on model fit. Results showed that the five factor model had a significantly better fit than the four factor model in which optimism and hope were forced to load on one factor.

In a third post-hoc analysis we investigated the possibility that a model in which, besides the five separate factors, a general factor is included that accounts for shared variance in all items would fit the data. Results showed that this model fit the data better than any other model tested and that there is a strong general factor that accounts for a large amount of the variance of the total bifactor model. Thus, we suggest that there is a general ‘outlook on future’ factor that underlies each of the items. Separately, there are four more specific factors namely treatment credibility, treatment expectancy, optimism and pessimism that each account for unique variance above this general factor. Hope however did not account for a substantial amount unique variance above the general factor.

Our findings are consistent with previous factor analyses that have shown hope and optimism to be related but distinct constructs [9,12]. Our study has extended these findings by demonstrating this in patients undergoing THA and TKA as well as by additionally considering treatment specific future oriented psychological constructs. Our results however slightly differ from Magaletta and Olivers [12] study because we found that the five factor model which included pessimism as a separate factor showed better model fit compared a four factor model in which all items of the LOT-R loaded on one factor. This could be a result of the use of the Dutch translation of the LOT-R which has shown to have a two-dimensional structure [31] Similar to previous studies we found that optimism and hope are positively related and that both of these are negatively related to pessimism [9,22].

Studying the conceptual overlap of psychological constructs seems to gain more importance. A reason for this is that many psychological measures have been developed in the last decades and all of them have individually shown to measure important constructs in medical care but considerable overlap may exist between these constructs (and measures), causing lack of conceptual clarity and confusion among researchers and care providers about which psychological measures to use in studies and daily practice. Therefore, studies investigating conceptual overlap or distinctiveness of these constructs within a medical care setting are important. Recently, two of such studies have been published. De Rooij et al [41] investigated the conceptual overlap between cognitive concepts in patients with chronic widespread pain and found that 16 different cognitive subscales could be reduced to three factors namely 1. negative emotional cognitions, 2.active cognitive coping and 3.control belief and expectations of chronicity. Campbell et al [42] studied the conceptual overlap of psychological constructs in low back pain patients and found that 20 subscales of psychological questionnaires could be reduced into four factors namely 1.pain-related distress, 2.cognitive coping, 3.causal beliefs and 4.perceptions of the future. Our study also addresses this issue; however we had a slightly different approach. De Rooij et al and Campbell et al performed factor analyses on a subscale level thereby aiming to identify the most complete though comprehensive set of cognitive (de Rooij) or psychological (Campbell) constructs. We however assessed whether individual item of questionnaires measuring the constructs of interest indeed load on the factors as intended by the developers of the subscale. Our approach therefore, may be seen as the first in a two-step approach in examining overlap between constructs. Once distinctive measurement has been established on an item level, a next step could then be assessing overlap between subscales as de Rooij and Campbell did.

### Strengths and limitations

A strength of this study is that the most widely accepted measurement instruments that aim to measure the included constructs were used, ensuring comparability to future CFA’s in other patient groups. A limitation of this study is the limited sample size for CFA’s, which made us decide to test our primary hypotheses on the complete sample of THA and THA patients. A multigroup analysis was only done to test for factorial invariance between the THA and TKA group. Although the FI models converged well and results suggest that strict invariance holds for our data, we do recognize that these analyses may be slightly underpowered and therefore these results should be interpreted with caution. Factorial invariance testing showed similar factorial structures in both groups implying that the constructs measured in this study have the same meaning in both patient groups, thereby suggesting generalizability of our results. Another limitation is the high percentage of missing responses on one of the items of the HS, which was caused by a printing error in the questionnaire. The WLMSV estimator in Mplus statistical software incorporates missing data by pairwise presence, though this is under the assumption that missing data are missing completely at random (MCAR). Because of the reason of the missing data and the fact that that participants with and without missing data (on item 6 of the HS) did not significantly differ on baseline characteristics we believe that the MCAR assumption may hold for our data.

Furthermore, we used Δχ^2^-test to compare models, which is controversial as the χ^2^ is influenced by sample size; therefore we also included other model fit statistics and based our conclusions on a combination of a-priori defined cut-points. CFA is a test of acceptance of a-priori defined models that are not data driven. A limitation of this method is however, that besides the theoretically plausible models tested in this study, there might be other models that show an even better fit to the data. Our results have furthermore not been validated in an external dataset we therefore encourage future research in TKA and THA and also in other patient groups.

## Conclusions and Implications

Based on the results of the current study and previous work we suggest that the constructs treatment expectancy, treatment credibility, hope, optimism and pessimism are distinguishable in THA and TKA patients. Posthoc, a bifactor model in which (besides the 5 separate factors) a general factor is hypothesized accounting for the commonality of the items showed a significantly better fit than the five factor model. All specific factors, except for the hope factor, showed to explain a substantial amount of variance beyond the general factor. Future studies should investigate the factorial structure of the CEQ. Our results may be valuable for the design of clinical studies aiming to measure one or more of these constructs as well as for the evaluation of interventions focussed on altering treatment expectancy which have been initiated lately by several groups [43–45]. As optimism and hope have been hypothesized to be relatively stable traits, it is necessary for researchers evaluating interventions aimed at altering treatment expectancy, to measure the possibly alterable treatment expectancy distinct from optimism and hope.

A next step in making these constructs of benefit for the patient undergoing THA and TKA is to investigate the relationships between these factors (e.g. Does optimism influence treatment specific expectancies?) and to find out which one or which combination of constructs predicts with more accuracy treatment outcomes after THA and TKA like pain, quality of life and physical well-being the best. In the future clinicians may use these constructs in addition to other tools, in order to identify patients with a high-risk for poor outcome in their decision for the type of intervention, either surgical or conservative.

## Supporting Information

S1 Fig Post-hoc model 1; four factor model in which the items of treatment credibility and treatment expectancy load on one factor. O1—O3 = LOT-R optimism items 1 to 3,P1—P3 = LOT-R reverse scored pessimism items 1 to 3,H1—H8 = ADHS hope items 1 to 8,C1—C3 = CEQ credibility items 1 to 3,E1—E3 = CEQ expectancy items 1 to 3 ovals represent latent factors, squares represent observed variables.
(TIF)

S2 Fig Post-hoc analysis 2, four factor model in which the optimism and hope items load on one factor. O1—O3 = LOT-R optimism items 1 to 3,P1—P3 = LOT-R reverse scored pessimism items 1 to 3,H1—H8 = ADHS hope items 1 to 8,C1—C3 = CEQ credibility items 1 to 3,E1—E3 = CEQ expectancy items 1 to 3 ovals represent latent factors, squares represent observed variables.
(TIF)

S3 Fig Post-hoc model 3; the bifactor model. O1—O3 = LOT-R optimism items 1 to 3,P1—P3 = LOT-R reverse scored pessimism items 1 to 3,H1—H8 = ADHS hope items 1 to 8,C1—C3 = CEQ credibility items 1 to 3,E1—E3 = CEQ expectancy items 1 to 3 ovals represent latent factors, squares represent observed variables.
(TIF)
