# Supplementary figures and images for: Can Optimism, Pessimism, Hope, Treatment Credibility and Treatment Expectancy Be Distinguished in Patients Undergoing Total Hip and Total Knee Arthroplasty?
Source: PLoS One. 2015 Jul 27;10(7):e0133730. doi: 10.1371/journal.pone.0133730 (PMC4516309; doi:10.1371/journal.pone.0133730)

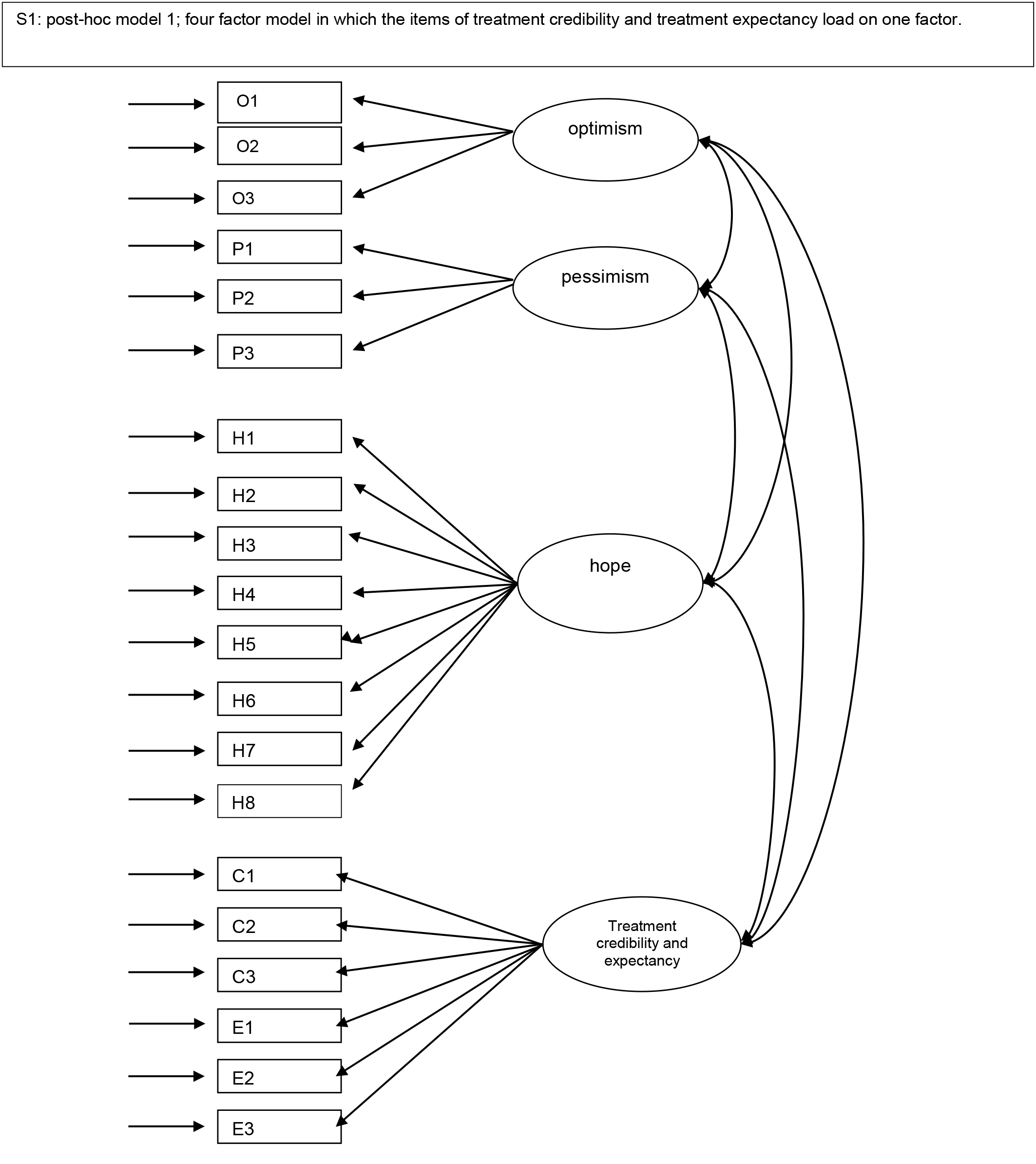

Supplement: S1 Fig — O1—O3 = LOT-R optimism items 1 to 3,P1—P3 = LOT-R reverse scored pessimism items 1 to 3,H1—H8 = ADHS hope items 1 to 8,C1—C3 = CEQ credibility items 1 to 3,E1—E3 = CEQ expectancy items 1 to 3 ovals represent latent factors, squares represent observed variables. (TIF) [file pone.0133730.s001.tif]

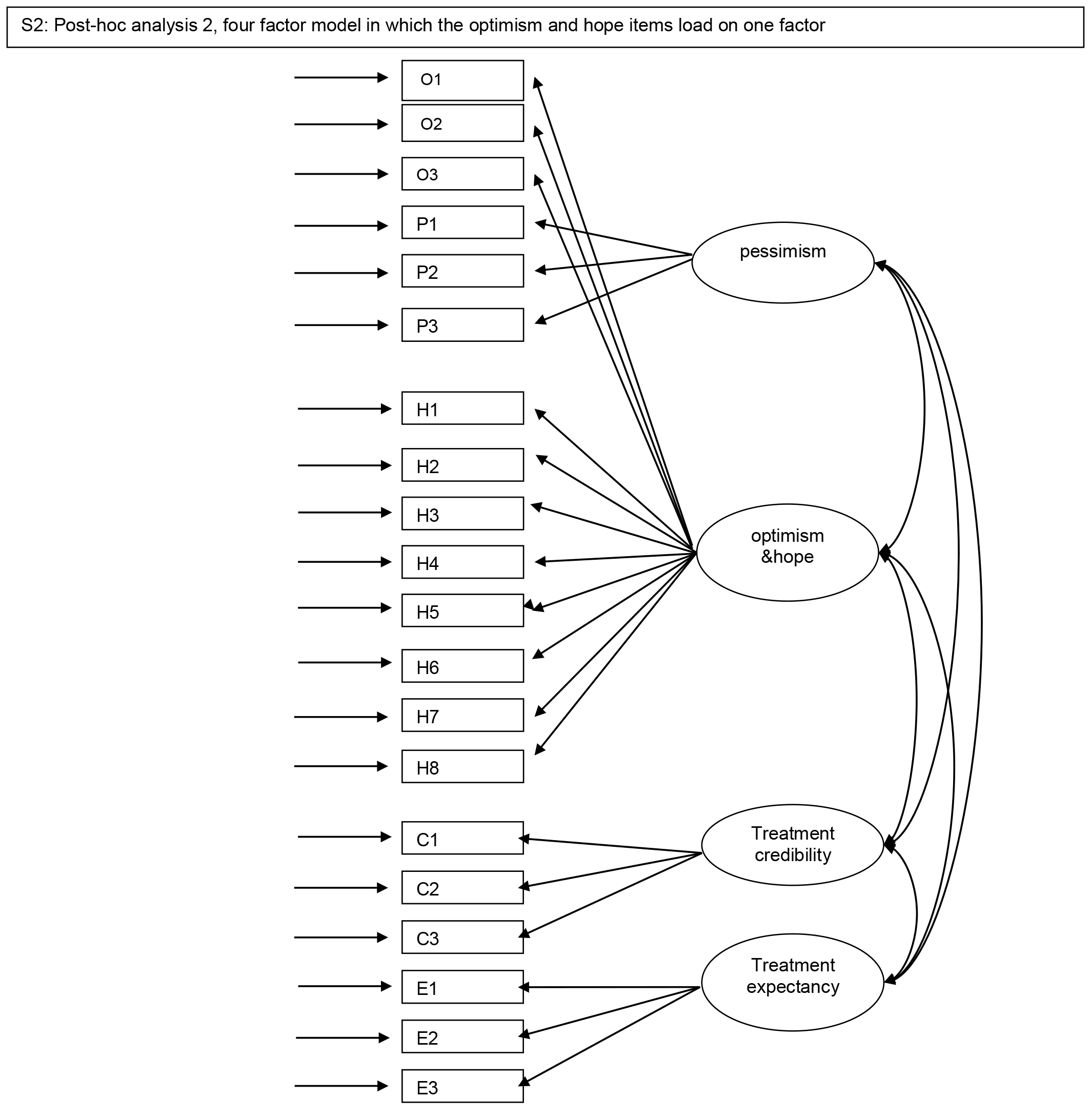

Supplement: S2 Fig — O1—O3 = LOT-R optimism items 1 to 3,P1—P3 = LOT-R reverse scored pessimism items 1 to 3,H1—H8 = ADHS hope items 1 to 8,C1—C3 = CEQ credibility items 1 to 3,E1—E3 = CEQ expectancy items 1 to 3 ovals represent latent factors, squares represent observed variables. (TIF) [file pone.0133730.s002.tif]

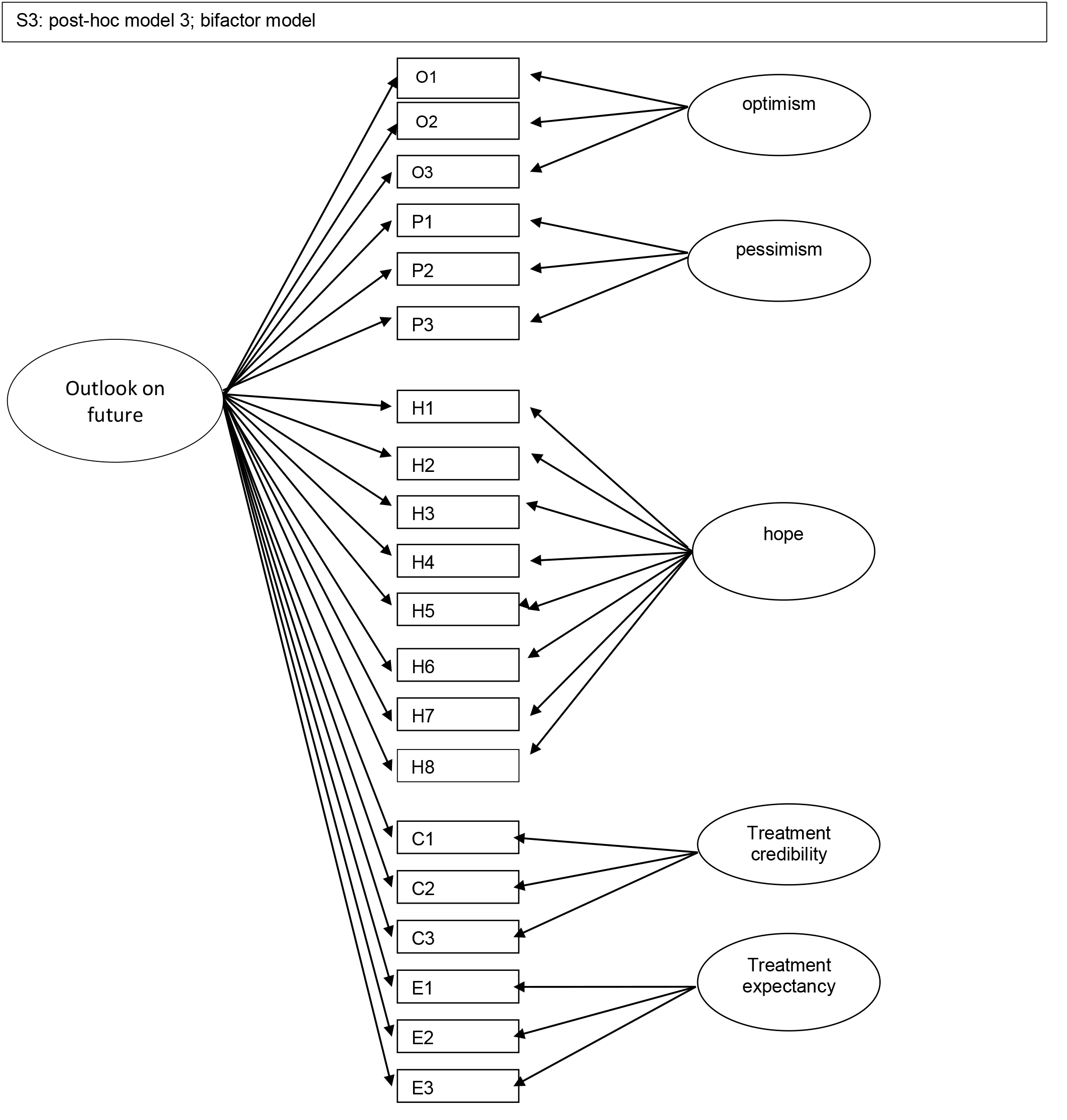

Supplement: S3 Fig — O1—O3 = LOT-R optimism items 1 to 3,P1—P3 = LOT-R reverse scored pessimism items 1 to 3,H1—H8 = ADHS hope items 1 to 8,C1—C3 = CEQ credibility items 1 to 3,E1—E3 = CEQ expectancy items 1 to 3 ovals represent latent factors, squares represent observed variables. (TIF) [file pone.0133730.s003.tif]
